# Characterization of Immune Response towards Generation of Universal Anti-HA-Stalk Antibodies after Immunization of Broiler Hens with Triple H5N1/NA-HA-M1 VLPs

**DOI:** 10.3390/v14040730

**Published:** 2022-03-30

**Authors:** Beata Gromadzka, Milena Chraniuk, Lilit Hovhannisyan, Karolina Uranowska, Bogusław Szewczyk, Magdalena Narajczyk, Mirosława Panasiuk

**Affiliations:** 1 NanoExpo sp.zo.o, Kładki 24/54, 80-822 Gdansk, Poland; m.chraniuk@ibmm.pl (M.C.); l.hovhannisyan@ibmm.pl (L.H.); panasiukmir@gmail.com (M.P.); 2 Department of In Vitro Studies, Institute of Biotechnology and Molecular Medicine, Kampinoska 25, 80-180 Gdansk, Poland; 3 Department of Recombinant Vaccines, Intercollegiate Faculty of Biotechnology, University of Gdansk, Abrahama 58, 80-307 Gdansk, Poland; uranowska.karolina@gmail.com (K.U.); boguslaw.szewczyk@biotech.ug.edu.pl (B.S.); 4 Laboratory of Electron Microscopy, Faculty of Biology, University of Gdansk, Wita Stwosza 59, 80-308 Gdansk, Poland; magdalena.narajczyk@biol.ug.edu.pl

**Keywords:** influenza virus, H5N1 triple VLPs, HA-stalk, universal anti-HA-stalk antibodies

## Abstract

(1) Background: Avian influenza viruses (AIVs) promptly evade preexisting immunity by constantly altering the immunodominant neutralizing antibody epitopes (antigenic drift) or by procuring new envelope serotypes (antigenic shift). As a consequence, the majority of antibodies elicited by infection or vaccination protect only against closely related strains. The immunodominance of the globular head of the main glycoprotein has been shown to mask the immunogenicity of the conserved regions located within the hemagglutinin (HA) protein. It has been shown that the broadly neutralizing universal antibodies recognize the HA2 domain in headless hemagglutinin (HA-stalk). Therefore, the HA-stalk is a highly conserved antigen, which makes it a good candidate to be used in universal vaccine development against AIVs. (2) Methods: Sf9 insect cells were used to produce triple H5N1/NA-HA-M1 influenza virus-like particles (VLPs) via co-expression of neuraminidase, hemagglutinin and matrix proteins from a tricistronic expression cassette. Purified influenza VLPs were used to immunize broiler hens. An in-depth characterization of the immune response was performed with an emphasis on the pool of elicited universal antibodies. (3) Results: Our findings suggest, that after vaccination with triple H5N1/NA-HA-M1 VLPs, hens generate a pool of broad-spectrum universal anti-HA-stalk antibodies. Furthermore, these universal antibodies are able to recognize the mammalian-derived HA-stalk recombinant proteins from homologous H5N1 and heterologous H7N9 AIVs as well as from the heterosubtypic human H1N1 influenza strain. (4) Conclusions: Our findings may suggest that highly pathogenic avian influenza H5 HA protein contain functional epitopes that are attractive targets for the generation of broad-spectrum antibodies against AIVs in their native hosts.

## 1. Introduction

Avian influenza virus (AIV) belongs to the genus Alphainfluenzavirus in the family Orthomyxoviridae (International Committee on Taxonomy of Viruses, 2018). AIVs are divided into subtypes based on the antigenic differences found in the two major surface glycoproteins—hemagglutinin (HA) and neuraminidase (NA) [1]. Currently, 16 HA subtypes (H1–H16) and 9 NA subtypes (N1–N9) in different combinations have been found in avian populations [2]. Moreover, the AIVs are classified as highly pathogenic avian influenza (HPAI) (H5 and H7 subtypes) and low pathogenic avian influenza (LPAI) (includes the rest of the subtypes) [3] viruses. Thus far, vaccination is the only effective method to control the burden of AIV, and in most cases, the inactivated form of the virus is utilized for vaccine preparation [4]. However, the inactivated vaccines arise further complications, including the inability of inducing a strong immune response. Moreover, it is difficult to differentiate between vaccinated and infected birds [5,6]. Later, to avoid these problems, the subunit vaccines containing HPAI virus HA protein were developed, which are currently in use in some countries. 

The technology of virus-like particles (VLPs) paved a new road in vaccinology when they were used for vaccine formulation. VLPs are self-assembled viral protein complexes whose structures closely mimic their parent viruses in conformation and organization [7]. VLPs are produced using a recombinant plasmid containing the coding region of the structural protein of a given virus [8]. The produced VLPs lack the viral genetic material, which consequently makes them a safe alternative to inactivated or attenuated viruses as they are incapable of being infectious and pathogenic [9]. VLPs of influenza viruses consisting of various combinations of the M1, M2, HA and NA proteins have been produced and assayed for their immunogenicity [10]. The HA, NA and M2 are shown to be strongly antigenic and, hence, are an important component of AIV VLP vaccines. Moreover, the M1 protein is responsible for the symmetry of the AIV VLPs. It has been shown that the VLPs are able to induce a higher immune response than the subunit vaccines [9]. Moreover, VLPs can be designed to specifically elicit an immune response based on the antigen used. Thus far, such VLPs have been produced based on AIV M1 protein [11]. Because of their immunogenic abilities, VLPs can interact with the immune system. By mimicking the role of an “antigen-presenting cell” they can interact with dendritic cells (DCs) via pattern recognition receptors (PRRs) on the surface of DCs [12,13]. After being engulfed by the DCs, VLPs are able to induce an adaptive immune response, which is stimulated by post-translational modifications of the VLPs. One of the most important modifications is the glycosylation of VLPs. Since the DCs carry glycan recognizing receptors, the glycosylated VLPs interact with them and get into the DCs. This highlights the importance of the expression system used since it is not always possible to acquire the desired post-translational modifications [11]. Although prokaryotic expression systems are widely used to produce VLPs, they are not able to make post-translational modifications. This lowers the immunogenic abilities of VLPs, making them not very efficient. In contrast to this, yeast, insect and mammalian expression systems are extensively used to create AIV VLPs with post-translational modifications. Moreover, recently, it has been shown that AIV VLPs produced in plants are also able to carry post-translational modifications [14]. The AIV VLP-based vaccine production is based on HA and NA proteins as the antigens, which are targeted by the antibodies against AIVs [15]. It has been demonstrated, that the humoral immune response is induced against the globular head of the HA protein. However, the antibody-binding sites on the HA are highly variable, which impairs the binding of the neutralizing antibodies [16]. Although these HA and NA antigens meet the criteria for inducing a robust antibody production, the dominant pool of antibodies produced does not target a conserved region on the AIVs and therefore, is not broadly protective. No data deeply characterizes the immune response in hens to describe the full repertoire of antibodies that may target the potential universal epitopes. 

Most strategies for designing AIV vaccines rely on the main glycoprotein of the influenza virus. HA is an essential multifunctional protein for virus pathogenicity. The HA protein allows for the binding of the virus to the host cell receptor and is considered to be the primary antigen that induces the neutralizing antibodies during infection [17]. Each HA monomer is synthesized as an immature precursor protein, HA0. The cleavage of HA0 protein into HA1 and HA2 subunits is crucial for viral infectivity [18,19]. The virus entry into the cell by endocytosis is initiated when the HA1 subunit binds to the sialic acid receptors on the cell surface. Later, because of the conformational change of the HA2 subunit, which is bound to the HA1 by disulfide bonds, the virus can fuse with the cell membrane [17]. The highly variable HA1 subunit (forming the globular head) contains major neutralizing epitopes. In contrast to this, the HA2 (containing most of the stalk domain) is conserved and contains only a few neutralizing epitopes which are cross-reactive [20,21]. These characteristics make the HA protein a preferable target for AIV vaccine development. All the universal neutralizing antibodies, such as the described FI6, until now, recognize the long alpha-helix (LAH) of the HA2 subunit within the HA-stalk antigen [22]. 

Hens are highly susceptible to the H5N1 HPAI virus infection and play a critical role in the spread of the virus. Chickens provide a natural model to determine the contributions of HA1 and HA2 subunits of the H5N1 HPAI virus for the induction of neutralizing antibody production and protection. There are several studies that use the intact HA protein of the H5N1 HPAI virus to produce viral vector vaccines for chickens [23,24]. To address the most relevant animal model for this study, we have chosen the natural host broiler hens instead of the specific-pathogen-free (SPF) chicken model. Here, we show for the first time, to our knowledge, a trivalent VLP vaccine that is able to elicit not only the immunodominant HA head neutralizing antibodies but also a set of universal antibodies that are able to recognize the HA-stalk antigen from both HA groups. 

## 2. Materials and Methods

### 2.1. Materials

Antibodies: Anti-H5N1 virus A/Ck/Scot/59 polyclonal chicken antibodies (cat. No RAA7002, Animal Health and Veterinary Laboratories Agency, Weybridge, UK); anti-H5N2 virus A/Ost/Den/72420/96 polyclonal chicken antibodies (cat. No RAA7003, Animal Health and Veterinary Laboratories Agency, Weybridge, UK); anti-M1 influenza mouse monoclonal (cat. No ab22396, Abcam Inc., Waltham, MA USA); anti-avian influenza A neuraminidase antibody (cat. No ab21304, Abcam Inc., Waltham, MA USA); anti-H1N1 mouse monoclonal antibodies (cat. No 11048-MM08, SinoBiological Inc., Beijing, China); FI6—highly specific humanized synthetic universal neutralizing monoclonal antibodies selected from plasma cells that bind group 1 and 2 influenza A HA (kind gift from Dr. Krzysztof Lacek and Alfredo Nicosia); anti-HA H5N1 mouse monoclonal antibodies (cat. No 11048-MM06, SinoBiological Inc., Beijing, China); anti-HA H3N2 mouse monoclonal antibodies (cat. No 11056-MM03, SinoBiological Inc., Beijing, China); anti-HA H7N9 mouse monoclonal antibodies (cat. No 11082-MM04, SinoBiological Inc., Beijing, China).

Peptides: LAH peptide, representing the long alpha-helix within HA-stalk from H3 subtype: biotinylated RIQDLEKYVEDTKIDLWSYNAELLVALENQHTIDLTDSEMNKLFEKTRRQLRENA LAH peptide (synthesized by JPT Peptide Technologies, Berlin, Germany).

### 2.2. Methods

#### 2.2.1. Cells, Viruses 

Madin–Darby canine kidney cells (MDCK.2; cat. No CRL-2936, ATCC) and human embryonic kidney 293 cells (HEK293 cells; cat. No CRL-1573, ATCC) were cultured in Dulbecco’s Modified Eagle’s Medium (DMEM; cat. No 11965092, Gibco, Waltham, MA, USA), supplemented with a 10% heat-inactivated fetal bovine serum (FBS; cat. No 10082147, Gibco, Waltham, MA, USA), 2 mM L-glutamine (cat. No A2916801, Gibco, Waltham, MA, USA), antibiotic antimitotic solution 1× (cat. No 15240062, Gibco, Waltham, MA, USA), at 37 °C under 5% CO_2_. 

The AIV A/ostrich/Denmark/725/96 (H5N2) and A/turkey/Poland/95/1995 (H7N2) were kindly provided by the Department of Poultry Diseases, National Veterinary Research Institute, Pulawy, Poland. Inactivated H5N1 virus A/Ck/Scot/59 and inactivated H5N2 virus A/Ost/Den/72420/96 were purchased from the Animal Health and Veterinary Laboratories (Weybridge, UK). The human influenza virus (the pandemic human influenza A/Virginia/ATCC3/2009/H1N1 virus) from ATCC (cat. No VR-1738) was also used in this study. HPAI H5N1 A/swan/Poland 305-135V08-2006 RNA for cDNA synthesis was kindly provided by the Department of Poultry Diseases, National Veterinary Research Institute, Pulawy, Poland.

All the active influenza A viruses were propagated in MDCK.2 cells in the presence of 2 µg/mL TPCK (L-1-tosylamide-2-phenylethyl chloromethyl ketone)—trypsin (cat. No 227800050, Thermo Scientific, Waltham, MA, USA).

*Spodoptera frugiperda* insect cells (Sf9; cat. No 12659017, Gibco, Waltham, MA, USA) were cultured in synthetic HyClone SFX-Insect cell culture media (cat. No SH30278.01, Cytiva, Marlborough, MA, USA) supplemented with antibiotic antimitotic solution 1x (cat. No 15240062, Gibco, Waltham, MA, USA), at 27 °C. Cells were grown in suspension or adherent culture depending on the experiment. 

#### 2.2.2. Genetic Constructs (Tricistronic Plasmids and HA-Stalk Constructs)

The cDNA synthesis of the full length of HA, NA and M1 genes from the H5N1 A/swan/Poland 305-135V08-2006 strain of the HPAI virus was carried out using universal primers and the Improm-II reverse transcription system (cat. No A3800, Promega, Madison, WI USA). The full length of genes coding structural proteins was amplified using Taq polymerase (cat. No 1201-200, A&A Biotechnology, Gdansk, Poland). The HA gene was amplified using primers For (5′- ATAGGATC CAAAATAGTGCTTCTTTTTGC-3′) and Rev (5′-AATACTATGACTCTGAACTCACAA ATTT-3′). The NA gene was amplified using primers For (5′- CTCGAGAGCAAAAGCAGGAGTTCAAA ATG-3′) and Rev (5′- AAGCTTCTACTTGTCAATGGTGAATGG-3′). The M1 gene was amplified using primers For (5′- GAATTC AGCAAAAGCAGGTAGATGTTG-3′) and Rev (5′- GGAGTAAAAAACTACCTTGTTTCTACTGAATTC-3′). The 1700 bp (HA), 1400 bp (NA) and 1000 bp (M1) PCR products were ligated as blunt fragments into a pGem-Teasy plasmid (Invitrogen, Waltham, MA, USA), followed by sequencing and subcloning into the pFastBac1 vector downstream of the ACMPV polyhedrin promoter. For pHA BamHI and SpeI, for pNA XhoI and HindIII, for pM1 EcoRI, recognition sites were used. All enzymes were purchased from Thermo Scientific (USA).

Tricistronic construct: First, the bacmid transfer vector expressing HA and M1 genes (pHA/M1) was obtained by subcloning the DNA fragment of pM1 plasmid containing M1 under the polyhedrin promoter, using SnaBI and HpaI restriction enzymes into the HpaI site of pHA plasmid. A bacmid transfer vector expressing all three structural proteins of the influenza virus (pNA/HA/M1) was constructed by subcloning the bicistronic cassette containing the HA and M1 genes from the pHA/M1 plasmid using SnaBI and AvrII restriction enzymes in the HpaI and AvrII sites of pNA plasmid. 

HA-stalk: All HA-stalk sequences were designed as headless, lacking the HA1 domain of hemagglutinin. To preserve proper conformation according to Steel et al., a glycine linker flanked with cysteines replaced the head region of the HA1 domain [25,26]. Synthetic genes of HA-stalk from H5N1 were cloned as EcoRI, NotI fragments into the pFastBac1 vector (Invitrogen, USA). Additionally, synthetic genes of HA-stalk from H5N1, H1N1 and H7N9 were cloned as BamHI and EcoRV fragments into the pcDNA3.1 vector (Invitrogen, USA).

#### 2.2.3. Recombinant Baculoviruses

Recombinant bacmids containing tricistronic expression cassettes and the HA-stalk coding sequence were generated using the Bac-to-Bac system (cat. No 10359-016, Thermo Fisher Scientific, Waltham, MA, USA). Briefly, *Escherichia coli* DH10 Bac competent cells were transformed with the recombinant pFastBac vector (pNA/HA/M1; pHA-stalk H5N1). The isolated bacmid DNA was purified and transfected into Sf9 cells using Lipofectamine (cat. No 18292011, Invitrogen, USA). After 6 days, recombinant baculoviruses (rBVs) were harvested, amplified, and titrated using the plaque assay method.

#### 2.2.4. VLP Production and Purification 

To produce influenza VLPs, Sf9 cells were infected with recombinant baculovirus at an MOI of 5 and harvested 96 h post-infection. The supernatant was collected after centrifugation in a microfuge at 8500 rpm for 10 min followed by ultracentrifugation for 2 h at 4 °C, 82,000× *g*. Concentrated VLPs were resuspended in NTE buffer containing EDTA and then purified on a 10%–60% sucrose gradient for 16 h at 4 °C, 82,000× *g*. Purified VLPs were further concentrated through ultracentrifugation for 2 h at 4 °C, 82,000× *g*. The proteins of each fraction were analyzed by SDS-PAGE and Western analysis. Purified influenza VLPs and viruses were visualized using transmission electron microscopy (TEM) and stored at 4 °C for further analysis. 

#### 2.2.5. SDS-PAGE and Western Blot Analysis 

For SDS-PAGE, proteins/VLPs were dissolved in the denaturing loading 4X Bolt™ LDS Sample Buffer (cat. No B0007, Thermo Fisher, Waltham, MA, USA) and heated for 10 min at 82 °C. Aliquots of 40 μL of each sample were loaded on 10%–20% precast Wedge Well gel (cat. No XP04202BOX, Thermo Fisher Scientific, Waltham, MA, USA) and run at a constant voltage of 200 V. Polyacrylamide gel was stained with Coomassie brilliant blue solution (cat. No 112553, Sigma-Aldrich, St. Louis, MO, USA). To perform a Western blot analysis, a semi-dry immunotransfer was performed using a Bio-Rad kit (cat. No 1704272, Bio-Rad, Warsaw, Poland). After the transfer, PVDF membranes were blocked for 1 h in a 5% semi-skimmed milk solution and incubated for 1 h with primary antibodies (anti-H5N1; anti-NA; anti-HA; anti-M1) in a 5% milk solution. Next, membranes were washed and incubated with appropriate secondary HRP/AP-conjugated antibody solution. The reaction was developed with ECL Plus Chemiluminescent substrate for horseradish peroxidase (cat. No 34580, Thermo Scientific, Waltham, MA, USA) or NBT/BCIP substrates (cat. No 34042, Thermo Scientific, Waltham, MA, USA).

#### 2.2.6. Transmission Electron Microscopy 

Influenza VLPs purified from the supernatant of infected Sf9 cells were diluted in TM buffer (10 mM Tris-HCl, pH 7.4 and 10 mM MgCl_2_ buffer). VLPs and viral particles were adsorbed onto carbon-coated grids, stained with 2% uranyl acetate, and examined immediately in a Philips CM 100 electron microscope. 

#### 2.2.7. Neuraminidase Activity Assay 

The neuraminidase activity was determined by neuraminidase enzyme assay using fetuin as a substrate of sialic acid cleavage (Amplex Red Neuraminidase (sialidase) assay kit, cat. No A22178, Life Technologies, Waltham, MA, USA). An aliquot of influenza VLPs and positive control virus were incubated with fetuin for 16 h at 37 °C. The amount of sialic acid released was determined chemically with thiobarbituric acid which produces a pink color in proportion to the amount of free sialic acid, which was measured by spectrophotometry at 560 nm. 

#### 2.2.8. Hemagglutinin Assay

The hemagglutination test was performed according to the standard protocol (PIWET, Poland). Briefly, a twofold dilution of the H5 antigens in a form of VLPs was incubated with 1% chicken erythrocytes for 30 min at room temperature. The positive control was the inactivated H5N1 virus A/Ck/Scot/59 and inactivated H5N2 virus A/Ost/Den/72420/96 from the Animal Health and Veterinary Laboratories Agency (Weybridge, UK). The negative control was the medium collected from uninfected Sf9 cells.

#### 2.2.9. Immunization of Chickens

H5N1 avian influenza VLPs obtained in the baculovirus system in insect cells were used to immunize chickens. A group of six 3 week old broiler hens (Ross 308) were housed in cages in an experimental poultry house under standard commercial conditions. The vaccine was administered intramuscularly into the breast muscle with a dose of 200 µL of the vaccine preparation containing 15 µg of VLPs and incomplete Freund’s adjuvant (ICF). The second group of six 3 week old broiler hens, vaccinated with 1:1 PBS/ICF, served as the control. After 17 days, a booster shot was performed with the same dose of the vaccine. Blood was collected for analysis on days 0, 17, 35 and 41. The immune response of the chickens was tested with ELISA and hemagglutination inhibition (HI) tests. The experiment was approved by the II Local Ethical Committee for Animal Experiments at the Medical University of Warsaw, permit number 17/2009. All efforts were made to minimize animal suffering. At the end of the experiment, the birds were humanely euthanized by decapitation.

#### 2.2.10. Hemagglutinin Inhibition 

Sera collected from triple H5N1/NA-HA-M1 VLP vaccinated birds on day 41 were pooled and tested in the hemagglutinin inhibition (HI) test. The HI test was performed according to a standard procedure (OIE, 2015). Briefly, 25 µL of sera in serial twofold dilutions were incubated for 25 min with four HA units of the inactivated H5N1 virus A/Ck/Scot/59 from the Animal Health and Veterinary Laboratories Agency (Weybridge, UK). Next, 25 µL of suspension of 1% hen erythrocytes was added and incubated for 30 min at room temperature. The HI titer was determined as the reciprocal of the highest dilution in which hemagglutination is inhibited. Samples were assigned as positive when their titer was ≥16. The negative control was the serum of hens vaccinated with a 1:1 PBS/ICF solution. H5N1 A/Ck/Scot/59 and H5N2 A/Ost/Den/72420/96 reference antibodies were used as a positive control. 

#### 2.2.11. ELISA 

##### Quantification of the Immune Response to Vaccination with Triple H5N1/NA-HA-M1 VLPs in Hens

The ELISA test was used to detect and quantify the immune response toward triple H5N1/NA-HA-M1 VLP vaccination in hens. A 96-well plate (Greiner Microlon High-Binding, clear) was coated with the 50 ng/well reference antigen inactivated H5N1 virus, purchased from the Animal Health and Veterinary Laboratories Agency reference laboratory (Weybridge, UK). The coated plate was incubated overnight at 4 °C. Then, the plate was washed 4 × 5 min with 200 µL/well of washing buffer (PBS/0.05%Tween20), blocked for 1 h at 37 °C with 250 µL/well of blocking buffer (3%BSA/PBS/0.05%Tween20) and washed as previously. Next, 100 µL/well of chicken sera diluted 1:300 from immunized hens collected on days 0, 17, 34, 41 after the first immunization was added and incubated for 1 h at 37 °C, and the plate was washed as previously. Next, 100 µL/well of AP-conjugated goat anti-chicken IgY antibodies (cat. No sc-2429, Santa Cruz Biotechnology, Dallas, TX, USA) (1:2000 in 3%BSA/PBS/0.05%Tween20) were added and incubated for 1 h at room temperature. Finally, following the last plate-washing step (6 × 5 min with 200 µL/well) 100 µL/well of TMB chromogen solution (cat No. 002023, Invitrogen, USA) was added. The reaction was stopped with 0.5 M sulphuric acid. The signal intensity was measured at 450 nm using a plate reader (TECAN, San Jose, CA, USA).

##### End-Point Titration of Sera from Chickens Immunized with Triple H5N1/NA-HA-M1 VLPs

Sera from immunized broiler hens were collected and pooled on day 41 after immunization. A 96-well ELISA plate (Greiner Microlon High-Binding, clear) was coated with 50 ng/well reference antigen inactivated H5N1 virus, purchased from the Animal Health and Veterinary Laboratories Agency reference laboratory (Weybridge, UK). The coated plate was incubated overnight at 4 °C. Then, the plate was washed 4 × 5 min with 200 µL/well of washing buffer (PBS/0.05%Tween20), blocked for 1 h at 37 °C with 250 µL/well of blocking buffer (3%BSA/PBS/0.05%Tween20) and washed as previously. After washing, serial dilutions of pooled chicken sera (in 3%BSA/PBS/0.05%Tween20) were added to the wells and incubated for 1 h at room temperature. Serial dilutions (in 3%BSA/PBS/0.05%Tween20) of chicken polyclonal antibody A/H5N1/HPAI from the Veterinary Laboratories Agency (Weybridge, UK) served as a positive control. After incubation, the plate was washed as previously, and a secondary antibody solution (AP-conjugated goat anti-chicken IgY antibodies; cat. No sc-2429, Santa Cruz Biotechnology, Dalas, TX USA) in 3%BSA/PBS/0.05%Tween20 was used for detection. Finally, following the last plate-washing step (6 × 5 min with 200 µL/well), 100 µL/well of TMB chromogen solution (cat No. 002023, Invitrogen, USA) was added. The reaction was stopped with 0.5 M sulphuric acid. The signal intensity was measured at 450 nm using a plate reader (TECAN, USA).

##### Cross-Reactivity of Heterosubtypic LAH of Human H3N2 HA2 Domain with Hen Sera after Vaccination with Triple H5N1/NA-HA-M1 VLPs by Peptide ELISA 

A 96-well streptavidin-coated ELISA plate (Pierce streptavidin high-binding capacity, clear) was coated with 100 µL/well of biotinylated RIQDLEKYVEDTKIDLWSYNAELLVALENQHTIDLTDSEMNKLFEKTRRQLRENA LAH peptide (synthesized by JPT Peptide Technologies) adjusted to 10 µg/mL. The coated plate was incubated for 2 h with shaking at room temperature. Then, the plate was washed 4 × 5 min with 200 µL/well of washing buffer (Tris-buffered saline pH 7.2/0.1%BSA/0.05%Tween20). Next, 100 µL/well of chicken sera diluted 1:300 from vaccinated hens was added. The plate was incubated for 2 h with shaking at room temperature and washed as previously. Next, 100 µL/well of HRP-conjugated goat anti-chicken IgY secondary antibody (cat. No sc-2428, Santa Cruz Biotechnology, Dallas, TX USA) was added, and the plate was incubated for 1 h at room temperature. Finally, following the last plate-washing step (6 × 5 min with 200 µL/well), 100 µL/well of TMB-substrate solution (cat. No 002023, SigmaFast; Sigma) was added. The reaction was stopped with 0.5 M sulphuric acid. The signal intensity was measured at 450 nm using a plate reader (TECAN).

#### 2.2.12. Characterization of HA-Stalk in Insect Cells via Immunohistochemical Assay with Anti-H1N1 Mab Antibodies or Universal FI6 Antibodies

Sf9 cells (8 × 10^5^) plated in a 2.5-cm culture dish in a synthetic SFX medium were infected with the H5N1 HA-stalk baculovirus at an MOI of 5. At 48 h post-infection, cells were washed with PBS and fixed with 70% cold methanol for 20 min. Next, fixed Sf9 cells were washed twice with PBS buffer and incubated for 1 h at room temperature with 500 µL/well of anti-H1N1 Mab antibodies diluted 1:1000 or with FI6 hMab antibodies diluted 1:1000 with 1% Tween 80 in PBS. Next, 500 µL/well of secondary antibodies—HRP-conjugated goat anti-mouse IgG (cat. No sc-2005, Santa Cruz Biotechnology, Dallas, TX USA) and HRP conjugated goat anti-human IgG (cat. No sc-2907, Santa Cruz Biotechnology, Dallas, TX USA), diluted 1:2000 in PBS and 1% Tween 80 was used. The plate was incubated for 1 h and washed 3 times with 1 mL of washing buffer (Tris-buffered saline pH 7.2/0.1%BSA/0.05%Tween80). The reaction was developed with the NovaRED substrate kit (cat. No SK-4800, Vector Laboratories, Burlingame, CA, USA). The cells were visualized using a Nikon PCM2000 confocal microscope. 

#### 2.2.13. Detection of Different HA-Stalk in Mammalian Cells via Immunohistochemical Assay with Hen Sera after Triple H5N1/NA-HA-M1 VLP Vaccination

HEK293 cells (8 × 10^5^) plated in a 2.5-cm culture dish in complete DMEM were transfected with pcDNA3.1 H1N1 HA-stalk, pcDNA3.1 H5N1 HA-stalk or pcDNA3.1 H7N9 HA-stalk using the JetPrime transfection reagent according to the manufacturer’s instructions (PolyPlus, New York, NY, USA). Forty-eight hours post-transfection cells were washed with PBS, frozen at −70 °C for 15 min, and fixed with 4% paraformaldehyde. Next, cells were incubated with 500 µL/well of chicken sera from day 41 of the immunization experiment diluted 1:300 in PBS with 1% Tween 80. The plate was incubated for 1 h and washed 3 times with 1 mL of washing buffer (Tris-buffered saline pH 7.2/0.1%BSA/0.05%Tween80). Next, 500 µL/well of secondary antibodies—HRP-conjugated goat anti-chicken IgY antibodies (cat. No sc-2429, Santa Cruz Biotechnology, Dallas, TX USA), diluted 1:2000 in PBS and 1% Tween 80 was used. The plate was incubated for 1 h and washed 3 times with 1 mL of washing buffer (Tris-buffered saline pH 7.2/0.1%BSA/0.05%Tween80). The reaction was developed with the NovaRED substrate kit (Vector Laboratories, USA). The cells were visualized using a Nikon PCM2000 confocal microscope.

#### 2.2.14. Statistical Analysis

Collected data were analyzed and visualized using GraphPad Prism (GraphPad Software, San Diego, CA, USA) and Excel (Microsoft, Redmond, WA, USA). The analysis was carried out with the use of nonparametric tests due to the limited number of samples, which were insufficient to confirm the normal distribution of obtained results.

##### Statistical Analysis for Quantification of the Immune Response towards Vaccination with Triple H5N1/NA-HA-M1 VLPs in Hens 

Statistical analysis of antibody titers in sera collected at different time points was carried out in GraphPad Prism with the use of the nonparametric Kruskal–Wallis test (*p* = 0.05). In the next step, to control the false discovery rate, the Benjamini, Krieger and Yekutieli multiple comparison test (*p* = 0.05) was conducted. Each serum was tested in duplicates and average measurements of absorbance were used for the statistical analysis.

##### Statistical Analysis for Cross-Reactivity of Heterosubtypic LAH of Human H3N2 HA2 Domain with Hens’ Sera after Vaccination with Triple H5N1/NA-HA-M1 VLPs

A comparison of antibody titer for sera collected before and after vaccination was carried out with the use of a nonparametric Wilcoxon test (*p* = 0.05) for paired groups. Statistical analyses were conducted for sera collected from each hen and also between the groups of unvaccinated and vaccinated animals. Each serum was tested in duplicates in two technical repeats.

#### 2.2.15. Alignment of LAH Region Amino Acid Sequences of Different Influenza Strains

H5N1, H1N1, H7N9 and H3N2 HA proteins’ LAH region amino acid sequences (The National Center for Biotechnology Information, Influenza Virus database, accession numbers: ADA83041 A/Abakan/02/2009/(H1N1), AGL44438 A/Shanghai/02/2013 (H7N9), ABU50579 A/Texas/01/2007 (H3N2), EpiFlu database, accession number: EPI156789 A/swan/Poland 305-135V08-2006 (H5N1)) were compared with Geneious Prime software using the Geneious alignment algorithm.

## 3. Results

### 3.1. Construction, Production and Purification of H5N1 Avian Influenza Triple H5N1/NA-HA-M1 VLPs

**Figure 1:**
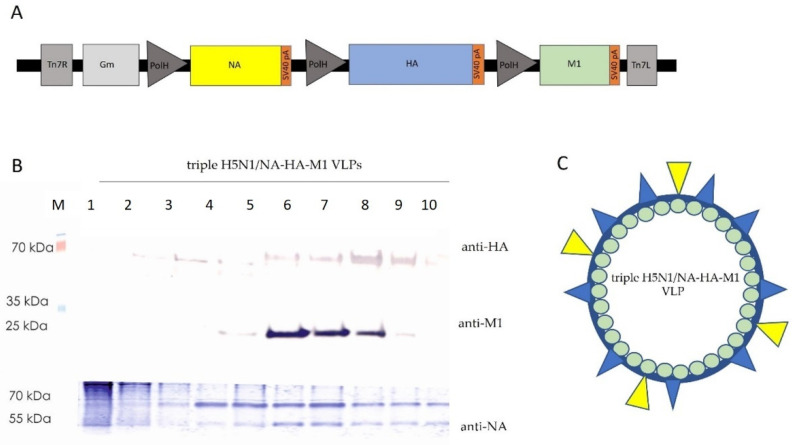
Production of H5N1 avian influenza triple H5N1/NA-HA-M1 VLPs. (**A**) Schematic representation of the tricistronic expression cassette. (**B**) Western blotting analysis of NA-HA-M1 VLPs purified on a 10%–60% sucrose gradient. Antibodies used for the detection of HA, NA and M1 proteins: mouse monoclonal anti-HA H5N1 antibodies, mouse monoclonal anti-M1 influenza antibodies and rabbit polyclonal anti-avian influenza A neuraminidase antibody. (**C**) Schematic representation of the predicted structure of triple H5N1/NA-HA-M1 VLPs. Structural proteins are color-coded according to the scheme of the expression cassette: NA—yellow, HA—blue, M1—green.

To achieve simultaneous expression of all three structural proteins of influenza (neuraminidase, hemagglutinin and matrix protein) from one expression vector, a tricistronic expression cassette was generated. Genes coding NA, HA and M1 were amplified by PCR from cDNA of the H5N1 A/swan/Poland 305-135V08-2006 strain of the HPAI virus. PCR products were ligated into the pFastBac1 vector downstream of the ACMPV polyhedrin promoter to obtain monocistronic constructs—pNA, pHA and pM1. Next, a bicistronic vector expressing HA and M1 genes (pHA/M1) was obtained by subcloning the DNA fragment of the pM1 plasmid containing M1 under the polyhedrin promoter into a plasmid. A bacmid transfer vector expressing all three structural proteins of the influenza virus (pNA/HA/M1) was constructed by subcloning the bicistronic cassette containing HA and M1 gene from the pHA/M1 plasmid into the pNA plasmid. A schematic representation of the tricistronic expression cassette is shown in Figure 1A and a detailed description of molecular cloning can be found in the methods section.

The final pNA/HA/M1 construct was then used to generate recombinant baculovirus using the Bac-to-Bac system. In order to produce influenza VLPs, Sf9 cells were infected with NA/HA/M1 recombinant baculovirus. The medium from infected cells containing VLPs was collected, concentrated and purified on a 10%–60 % sucrose gradient. The proteins of each fraction were analyzed by SDS-PAGE and Western blotting, confirming the presence of all three proteins (Figure 1B). The scheme of the predicted structure of triple H5N1/NA-HA-M1 VLPs is shown in Figure 1C. Additionally, the influenza A/H5N2 virus propagated in MCDK.2 cells was purified and visualized as a positive control using the same method. Purified triple H5N1/NA-HA-M1 VLPs were present in the same fractions of sucrose gradient as the virus, which suggests the proper assembly and stability of the VLPs and little to no deviation in shape, as compared to the virus (data not shown).

**Figure 2:**
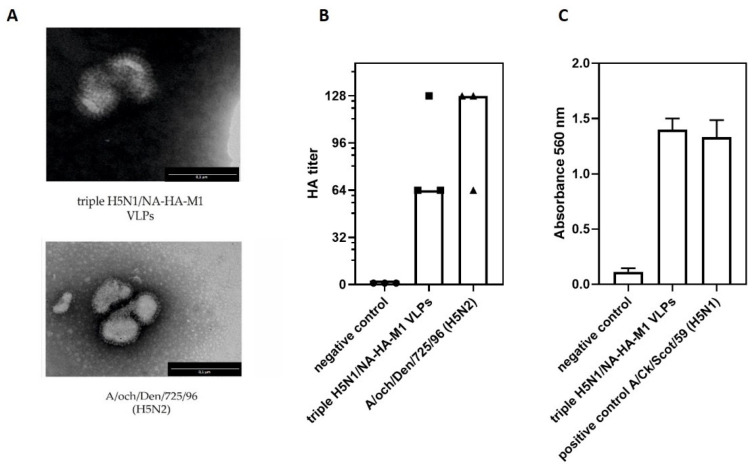
Characterization of H5N1 avian influenza virus triple H5N1/NA-HA-M1 VLPs. (**A**) Transmission electron microscopy of triple H5N1/NA-HA-M1 VLPs and influenza A/H5N2 virus. Scale bar = 1 µm is shown in the right bottom corner of the images. (**B**) Hemagglutination assay. The AIV A/ostrich/Denmark/725/96 (H5N2) was used as a positive control. The HA titer was determined as the reciprocal of the highest dilution with HA activity. Each dot represents HA titers obtained in each experiment. The bars represent the median values of obtained HA titers. (**C**) Neuraminidase activity assay. The AIV A/Ck/Scot/59 (H5N1) was used as a positive control. For each assay, the mean value from three independent experiments performed is presented. The mean A560 values and standard deviations are shown on the y-axis.

Purified triple H5N1/NA-HA-M1 VLPs were visualized using transmission electron microscopy, revealing a spherical structure similar to the AIV (Figure 2A). To confirm the proper functionality of the VLPs, the hemagglutination assay and the neuraminidase activity assay were performed. Obtained results show that both HA (Figure 2B) and NA (Figure 2C) that are present on the surface of VLPs were properly folded and fully functional. There were only small differences between the activity levels of proteins on the surface of VLPs and proteins present on the surface of the influenza virus used as a reference. Those results confirm the correct assembly of H5N1 avian influenza triple H5N1/NA-HA-M1 VLPs and their functionality. 

### 3.2. Immunization of Broiler Hens with H5N1 Avian Influenza Triple H5N1/NA-HA-M1 VLPs 

**Figure 3:**
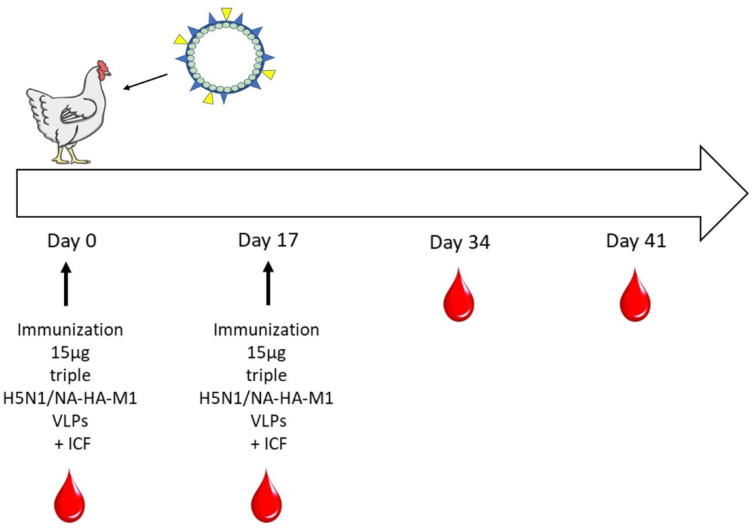
Schematic timeline of immunization of the broiler hens with triple H5N1/NA-HA-M1 VLPs. Red droplets represent the days of blood collection.

In the next part of this study, purified triple H5N1/NA-HA-M1 VLPs were used to immunize chickens. To assess the immune response to the potential vaccine in the animal model of the natural poultry industry, broiler hens were used. Birds were vaccinated intramuscularly into the breast muscle with 15 µg of VLPs and ICF. After 17 days, chickens were vaccinated again with the same formulation of the vaccine. Blood was collected for analysis at 0, 17, 34 and 41 days of the experiment. A schematic timeline of the immunization is shown in Figure 3.

### 3.3. Analysis of the Immune Response after Immunization with Triple H5N1/NA-HA-M1 VLPs

**Figure 4:**
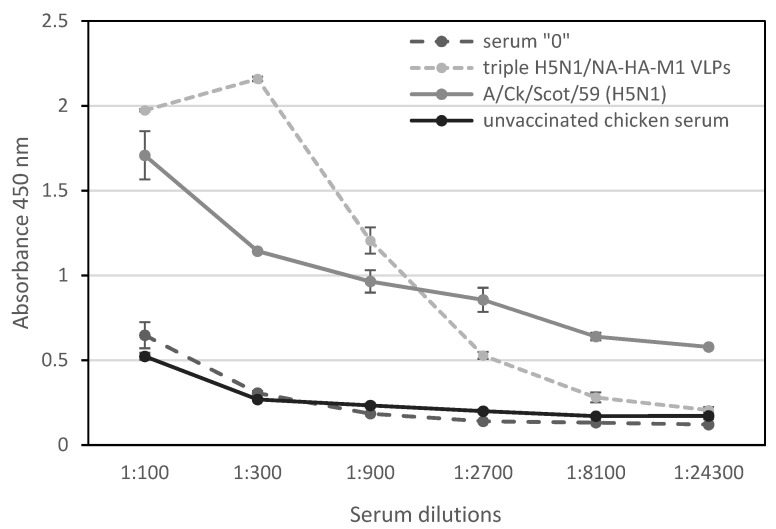
End-point titration of chicken sera after immunization with triple H5N1/NA-HA-M1 VLPs. ELISA plates were coated with reference antigen inactivated H5N1 virus. Serial dilutions of chicken A/H5N1/HPAI polyclonal antibodies served as a positive control. Chicken serum from day 0 and control chicken serum served as a background. The dilution factor of the pooled sera is shown on the x-axis. For each ELISA, the mean value from three independent experiments performed is presented. The mean A450 values and standard deviations are shown on the y-axis.

To assess the immune response to the potential vaccine in broiler hens, the end-point titration of sera via ELISA assay was performed. The end-point serum titrations show that the final antibody titer obtained after immunization with triple H5N1/NA-HA-M1 VLPs reached 1:2700 (Figure 4). The antibody titer was estimated as the serum concentration at which the binding was at least twice higher than the background.

**Figure 5:**
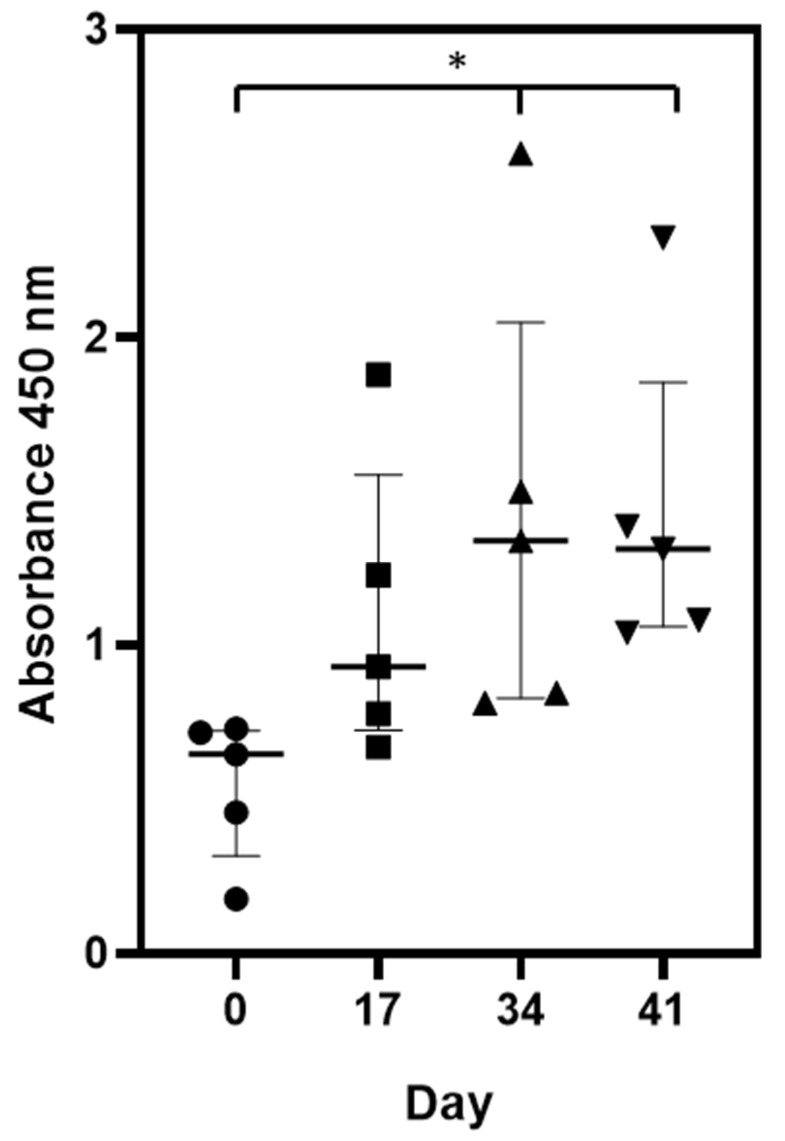
Dynamics of anti-H5N1 IgY level in sera of immunized chickens. Kinetics of antibody titer in chickens (*n* = 5) following prime and boost vaccinations with triple H5N1/NA-HA-M1 VLPs were measured via ELISA test. The median (thick line) is shown with the interquartile 25% and 75% range (narrow lines). The day of the serum collection is shown on the x-axis. The A450 values are shown on the y-axis. Statistical analysis was performed using the nonparametric Kruskal–Wallis test (*p* = 0.05) and Benjamini, Krieger and Yekutieli multiple comparison test (*p* = 0.05). Statistical differences were detected between days 0–41 and 0–34 (*p* = 0.014) and shown on the graph as a star symbol.

To further evaluate the humoral response of broiler hens to the immunization with triple H5N1/NA-HA-M1 VLPs, antibody production kinetics were evaluated (Figure 5). 

An analysis of antibody titers in blood collected at 0, 17, 34 and 41 days of the experiment revealed a steady increase in the humoral response after broiler immunization with triple H5N1/NA-HA-M1 VLPs. Robust production of the antibodies was detected after the first dose of the vaccine. The second dose of triple H5N1/NA-HA-M1 VLPs did not significantly increase the number of antibodies produced. 

**Figure 6:**
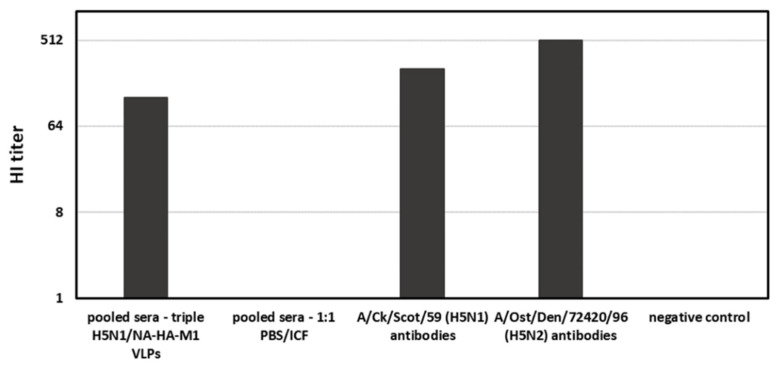
HI titer of pooled chicken sera collected after immunization with triple H5N1/NA-HA-M1 VLPs. H5N1 A/Ck/Scot/59 and H5N2 A/Ost/Den/72420/96 antibodies were used as a positive control. Sera from chickens vaccinated with PBS/ICF mixture served as a negative control. HI assay was performed in triplicates.

Additionally, to evaluate the efficacy of the vaccination, an HI assay was performed. The HI titer of pooled chicken sera reached the level of 128 HI (Figure 6). It is estimated that samples are positive when the HI titer reaches ≥ 16. As a positive control, inactivated H5N1 A/Ck/Scot/59 and H5N2 A/Ost/Den/72420/96 viruses were used. The PBS vaccinated hens did not show any HI activity. 

### 3.4. Construction of HA-Stalk Universal Influenza Antigen

**Figure 7:**
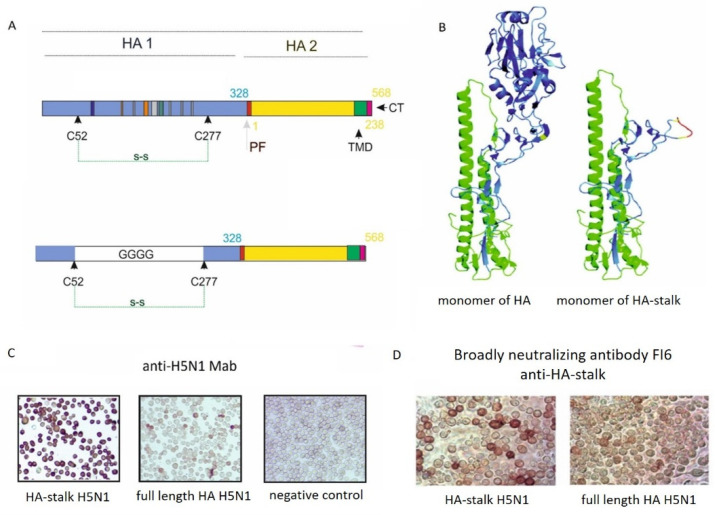
Construction and characterization of HA-stalk universal influenza antigen from homologous H5N1 HPAI virus strain. (**A**) Schematic representation of the full-length HA (top panel) and HA-stalk constructs (bottom panel). To obtain the HA-stalk construct, a glycine linker was added between cysteines in positions C52 and C277, replacing the head region of the HA1 domain. (**B**) Schematic representation of the predicted structure of HA monomer and HA-stalk monomer. (**C**) Expression of H5N1 HA-stalk in insect cells was confirmed by IPMA with anti-H5N1 monoclonal antibodies. Full-length HA from the H5N1 strain was used as a positive control. Cells infected with the wild type baculovirus were used as a negative control. Images were taken at ×10 magnification. (**D**) Reactivity of the HA-stalk antigen and full-length HA from H5N1 in the IPMA with broadly neutralizing universal FI6 human antibodies. HA-stalk and full-length HA from the H5N1 strain was detected in transfected insect cells. Images were taken at ×20 magnification.

To further characterize the repertoire of antibodies obtained after triple H5N1/NA-HA-M1 VLP vaccination in the hen model, their ability to bind the universal influenza antigens was tested. For this part of the study, the conserved stalk of the hemagglutinin protein (HA-stalk) was chosen as a universal influenza antigen. The recombinant HA-stalk antigen from the homologous H5N1 avian influenza strain (A/H5N1 HPAI virus) was designed according to Uranowska et al. and Krammer & Palese [27,28]. Briefly, to preserve proper conformation, a glycine linker was added between cysteines in positions C52 and C277, replacing the head region of the HA1 domain (Figure 7A,B). The expression of the HA-stalk was obtained in insect cells using the baculovirus expression system. Protein production was confirmed by IPMA (Figure 7C) and flow cytometry with anti-stalk antibodies (anti-H1N1 mouse monoclonal antibodies (cat. No 11048-MM08, Sino Biological Inc., Beijing, China)) (data not shown). Additionally, to confirm the proper folding of the HA-stalk antigen, the IPMA with broadly neutralizing universal FI6 human antibodies was performed (Figure 7D). It is worth mentioning, that the FI6 antibodies are described as synthetic antibodies capable of neutralizing multiple subtypes within group 1 or group 2 of HA and were isolated from the plasma cells of immune donors. Moreover, they were characterized as conformational and linear antibodies that bind the conserved HA region.

**Figure 8:**
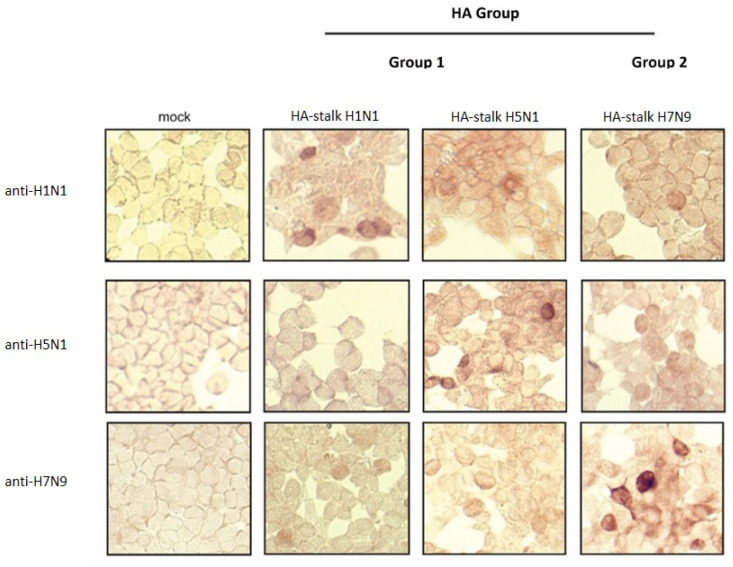
Expression of HA-stalk antigens from the 1 and 2 HA groups in mammalian cells. HEK293 cells were transfected with HA-stalk H1N1, H5N1 and H7N9 constructs. Protein expression was detected using different monoclonal antibodies specific for H1N1, H5N1 and H7N9 influenza strains. Non-transfected cells were used as a negative control. Images were taken at ×40 magnification.

Further, to confirm the broad spectrum of universal recognition of the obtained hens’ sera, HA-stalk constructs from both 1 and 2 HA groups were designed and produced in mammalian cells. The mammalian-derived HA-stalk recombinant protein from heterologous H7N9/Shanghai AIVs as well as from the heterosubtypic human pH1N1 influenza strain was designed with the same strategy as described above (Figure 7A). The synthetic sequences were cloned into mammalian expression vectors (pcDNA3.1; Invitrogen, USA) and used for transfection of HEK293 cells. To confirm the expression of different HA-stalk antigens, IPMA was performed. Each HA-stalk construct was detected with different monoclonal antibodies specific to the H1N1, H5N1, H9N2 and H7N9 influenza strains, as shown in Figure 8.

**Figure 9:**
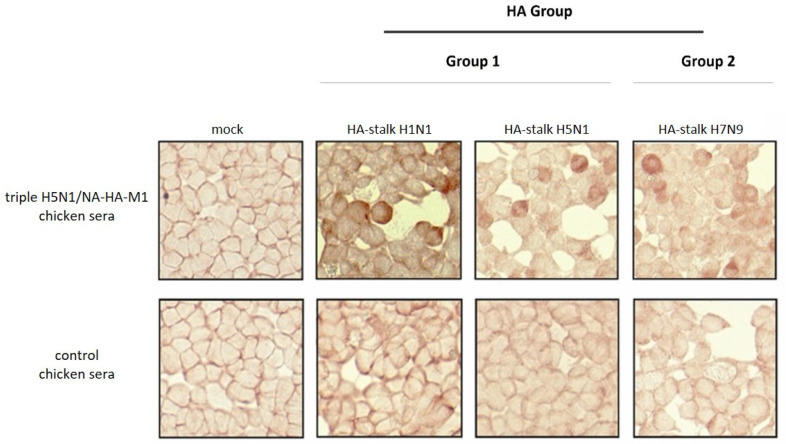
Cross-reactivity of chicken sera obtained after vaccination with triple H5N1/NA-HA-M1 VLPs with the HA-stalk antigens from the 1 and 2 HA groups. Detection of HA-stalk from H1N1, H5N1 and H7N9 strains was performed on transfected HEK293 cells. Sera from unvaccinated hens were used as a background. Non-transfected cells were used as a negative control. Images were taken at ×40 magnification.

Next, hens’ sera after vaccination with triple H5N1/NA-HA-M1 VLPs were used to detect mammalian-derived HA-stalk recombinant protein from heterologous H7N9/Shanghai AIVs as well as from heterosubtypic human pH1N1 and homologous H5N1 influenza strains. IPMA showed reactivity of the obtained sera with different HA-stalk universal antigens. These results indicate that immunization with triple H5N1/NA-HA-M1 VLPs was able to elicit a pool of universal antibodies (Figure 9).

**Figure 10:**
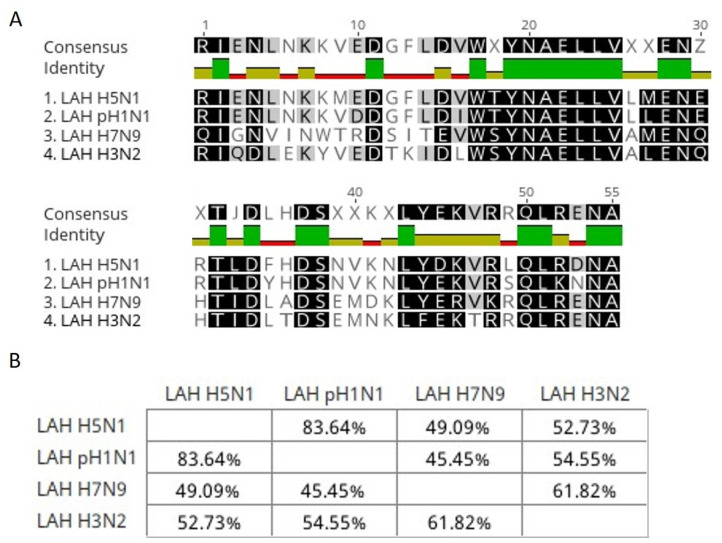
Alignment of amino acid sequences coding LAH regions from H5N1, pH1N1, H7N9 and H3N2 HA protein. (**A**) Alignment view with consensus sequence where the highest similarity is shown as a green colour. (**B**) Matrix showing the percentage of sequence identity between sequences.

The least variable region within HA-stalk is the LAH region. The alignment of amino acid sequences coding LAH regions from H5N1, pH1N1, H7N9 and H3N2 HA protein shows over a 45% sequence identity between them (Figure 10).

**Figure 11:**
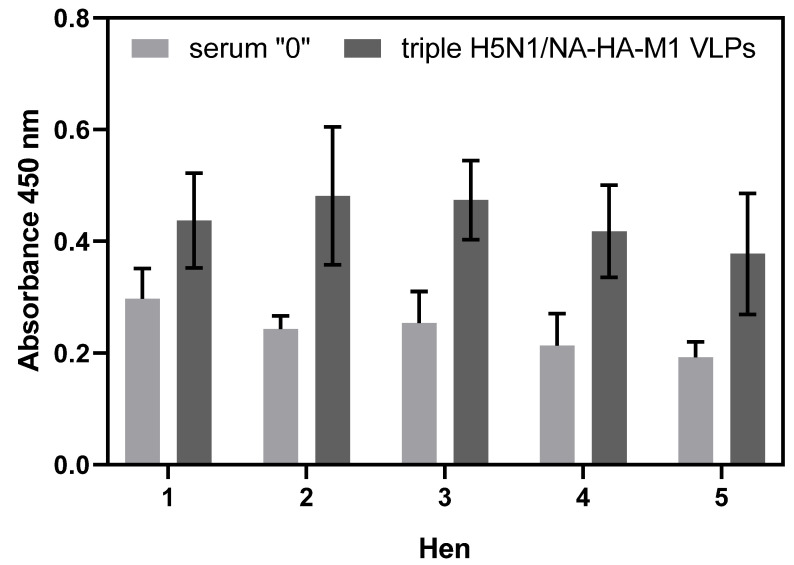
Cross-reactivity of chicken sera obtained after vaccination with triple H5N1/NA-HA-M1 VLPs with the LAH peptide from H3 from 2 HA group. The antibody titer in chickens (*n* = 5) before (gray) and after (black) vaccination with triple H5N1/NA-HA-M1 VLPs was measured via the peptide ELISA test. The mean OD values and standard deviations are shown on the y-axis. The tested chickens are shown on the x-axis. Statistical analysis was performed using a nonparametric Wilcoxon test (*p* = 0.05) for paired groups.

To further analyze the pool of universal antibodies elicited after vaccination of chickens with triple H5N1/NA-HA-M1 VLPs, peptide ELISA was performed on a plate coated with LAH peptide. Sera from day 0 and day 41 from five chickens were chosen to analyze the ability to bind LAH peptides. Obtained results suggest that sera from day 41 contained substantially higher amounts of universal IgY antibodies, specifically recognizing LAH epitopes (Figure 11). However, statistical analysis performed using a nonparametric test did not reveal statistically significant differences between samples obtained before and after vaccination in each chicken, nor between the grouped data. 

## 4. Discussion

The development of new technologies for the production of influenza vaccines is one of the biggest concerns of the poultry industry and public health. Vaccines containing complete, attenuated or inactivated viruses require the handling of infectious virus’ (or virus components) at BSL3 and/or BSL2-enhanced safety levels during the vaccine production process. Consequently, studies are focused on developing the next generation of vaccines containing recombinant antigens in the form of VLPs. These nanostructures seem to be a perfect vaccine since they not only imitate a native viral particle and do not contain genetic material, but also have the ability to stimulate both types of an immune response [28,29]. VLPs appear to be an ideal technological solution, as they are produced in cell cultures [30,31,32]. There are many ongoing studies on the immunogenicity, safety, and tolerability of VLP-based influenza vaccines. Influenza VLPs are formed by self-assembly incorporating structural proteins into particles by budding from the host cell membrane. Many articles describe HA, NA and M1 proteins in the form of extracellular particles that may also include additional influenza proteins, such as M2 [33]. These nanostructures include intact and biochemically active antigens required for the induction of both humoral and cellular immune responses [34]. 

In 2005, Pushko et al. described for the first time, that VLPs built with the HA, NA and M1 proteins of the H9N2 influenza virus induce protective immune responses in mice [35]. From that time, more than 60 different types of VLPs consisting of different influenza glycoproteins and structural proteins were described. Many of those extracellular particles have been obtained through different production strategies. Currently, using a variety of vectors and gene delivery techniques, influenza nanoparticles are produced in mammalian, insect and plant cell cultures [36,37,38].

Depending on the structural components of the influenza virus, different types of extracellular particles can be described: sub-viral particles composed of HA, NA and M2 proteins that lack the matrix protein M1; VLPs containing M1 protein and other influenza glycoproteins or M2 structural proteins; and capsid-like particles containing only the M1 protein, forming extracellular nanostructures. 

Diverse VLPs were obtained for different types of influenza (H5N1, H1N1, H7N9, H3N2) through different strategies. The most effective way of VLP production in insect cells is by the co-expression of influenza genes from one vector instead of co-infection with several baculoviruses expressing singular AIV genes (data not shown) [39,40]. The use of the polyhedrin promoter is more adequate than the p10 promoter, since the structural proteins NA, HA and M1 need to be expressed at comparable levels. Therefore, in this study, we designed a tricistronic expression cassette with three structural influenza proteins, each under a separate polyhedrin promoter. Thanks to the strategy, all genes were expressed simultaneously in each insect cell at high amounts, as confirmed in Western blotting analysis. Obtained triple H5N1/NA-HA-M1 VLPs were structural, as revealed by electron microscopy, and functionally comparable to a native virus, which served as a reference control. Electron micrographs revealed spherical VLPs with a diameter of approximately 120 nm, which are similar in size to influenza virions. VLPs comprised of an M1 protein core and a lipid envelope containing the HA and NA proteins were previously described [30,32,41,42]. Since two major glycoproteins are essential for the virus replication cycle, the characterization of their activity within VLPs was performed using hemagglutination assay and neuraminidase activity assay. Only small differences between the activity levels of two major glycoproteins were detected when compared to the reference H5N1 and H5N2 viruses. 

It is worth mentioning that the H5N1 subtype crossed the species barrier for the first time in 1997, and from that time still, there are several challenges in designing an effective vaccine. The phylogenetic characterization and sequence homology in the highly pathogenic H5N1 HA groups (viruses of 10 clades) are defined by the WHO/OIE/FAO H5N1 Evolution Working Group. Moreover, many of these clades have additional subclades and sub-subclades [43,44]. Currently, the main problem is that commercially available vaccines elicit a narrow strain-specific antibody response that neutralizes antigenically matched virus strains but fail to protect against drifted strains or newly emerging pandemic viruses. Protection against different influenza virus subtypes and variants requires the development of vaccines that can induce homo- and heterosubtypic immunity. To our knowledge, the VLP-based vaccines should target not only the major variable surface antigen of the virus—HA—but also more conserved internal antigens, such as the matrix M1 protein and/or M2 protein and HA-stalk antigen. Thus, to fully understand the immune response in the most relevant host model, broiler chickens were used in this study.

Our triple H5N1/NA-HA-M1 VLPs served as a surrogate model of highly pathogenic avian influenza strain to characterize the immune response in the natural model after vaccination. Hens were vaccinated twice with H5N1/NA-HA-M1 VLPs formulated with ICF. 

To evaluate the kinetics of humoral response of hens after vaccination with triple H5N1/NA-HA-M1 VLPs, direct ELISA with inactivated H5N1 virus was performed. The end-point titration of IgY antibodies after vaccination reached a high and comparable level to other data [44,45,46,47,48]. It is worthy of note that the triple H5N1 strain, which sequence was used to obtain triple H5N1/NA-HA-M1 VLPs was originated from clade 2.2.2. Additionally, the inactivated HPAI H5N1 virus used in the study belongs to clade 0. Our results indicate the homosubtypic cross-reactivity of produced IgY antibodies. The dynamics of IgY production revealed a steady increase in the antibodies. Moreover, the boost did not significantly increase the humoral response, which is comparable with the results obtained by other groups [49,50].

The efficacy of the triple H5N1/NA-HA-M1 VLP-based vaccine was evaluated via HI assay. The virus used in this assay was homosubtypic H5N1 from clade 0. The results show that the antigen in the form of VLPs from clade 2.2.2 elicits antibodies that possess the ability to inhibit the hemagglutination of homosubtypic antigen H5N1 on a high level of 128 HI units, which is above the cut-off of the protective level 40 HI unit [43]. In comparison with the results obtained for H5 avian influenza VLPs, described by Huang et al. [45], the obtained HI titers were much higher for homosubtypic strains from different clades. In comparison to the results by Choi et al. [51], our results showed much higher HI titers. 

Many studies describe the efficacy of the VLP immunization in order to define the HA dominant and NA dominant immune response in the chicken model. Here, we present a first-time in-depth characterization of the immune response toward a generation of universal anti-HA-stalk antibodies after vaccination of broiler hens with HPAI H5N1 VLPs. Our results suggest that after vaccination with triple H5N1/NA-HA-M1 VLPs, hens generate a pool of broad-spectrum universal anti-HA-stalk antibodies. To evaluate the immune response toward the headless hemagglutinin, we designed the HA-stalk antigens from different influenza strains. Our previous results by Uranowska et al. show the characterization of avian HA-stalk antigen as a potentially universal one [25]. Based on those findings, we designed additional HA-stalk antigens from heterosubtypic influenza strains. We performed experiments to assess if the mammalian-derived HA-stalk recombinant proteins from homologous H5N1 and heterologous H7N9 AIVs, as well as from the heterosubtypic human H1N1 influenza strain, can be detected by the sera obtained after vaccination with HPAI H5N1 VLPs. IPMA performed on human kidney cells expressing different HA-stalk antigens revealed the reactivity with the universal epitopes located within the HA-stalk region. This may suggest that HPAI H5 HA protein contains functional epitopes that are attractive targets for the generation of broad-spectrum antibodies against AIVs in their native hosts. 

LAH is the least variable region within the HA-stalk. It is considered to be the most universal epitope. To analyze whether the pool of universal antibodies elicited after vaccination of chickens with triple H5N1/NA-HA-M1 VLPs is able to recognize LAH, we performed an ELISA assay on plates coated with LAH peptide from the heterosubtypic H3N2 influenza strain. Our results suggest that hen sera from day 41 is cross-reactive with the heterosubtypic H3N2, and contains substantially higher amounts of universal IgY antibodies, specifically recognizing the LAH epitope. Statistical analyses of the results did not reveal significant differences between samples obtained before and after vaccination in each chicken or between grouped data. However, it is important to note that obtained absorbance values were at a low level due to the antibody dilutions used, which at the same time resulted in a decrease in the differences between the two types of tested samples. Moreover, a nonparametric test had to be used for the analysis. This test is characterized by lower statistical power. In the case of having a larger test group, more powerful parametric tests could be used for analysis, the use of which would give a chance to detect statistically significant differences. In the case of nonparametric tests, there is a risk of staying with a faulty hypothesis that the data groups are not statistically different.

Moreover, Kim et al. suggested that the chimeric cHA H9/H5N2 vaccination strategy provides robust protection against homologous, heterologous, and heterosubtypic viruses of both subtypes [52]. Furthermore, each HA1 and HA2-stalk-specific antibody response was sufficient to inhibit viral replication and protected the chimeric virus-immunized mice from lethal challenges with mouse-adapted H9N2, H5N2, or wild type HPAI H5N1 virus. This finding suggests that the HA2-stalk region of the H5 HA protein contains functional epitopes that are attractive targets for broad-spectrum H5 influenza viruses in both chicken and mouse models [52].

On the other hand, an H5N1 vaccine construct, incorporating either HA1 or HA2 subunit, may not provide protection against an HPAI virus challenge in chickens, according to Shirvani et al. [53]. Their results show that immunization with HA1 or HA2 alone neither induces serum neutralizing antibodies nor prevents death following the challenge. Immunization with the full-length HA protein is necessary for complete protection against the HPAI virus.

We strongly believe that further analysis towards the characterization of antibodies that are able to recognize the universal HA-stalk antigens is of great importance, thus helping to design a broad-spectrum vaccine against poultry threats.

## 5. Conclusions

Vaccination of broiler hens with triple HPAI H5N1 VLPs triggers the generation of broad-spectrum anti-HA-stalk antibodies. Furthermore, these universal antibodies are able to recognize the mammalian-derived form of HA-stalk recombinant proteins from homologous H5N1 and heterologous H7N9 AIVs as well as from the heterosubtypic human H1N1 influenza strain. To further evaluate the effectiveness of the prepared VLP vaccine, an in vivo challenge should be performed.

## Data Availability

The data presented in this study are available on request from the corresponding author.
